# Structured Prototype Learning with Feature Fusion for Sparse and Asynchronous Audio–Visual Depression Recognition

**DOI:** 10.3390/s26175580

**Published:** 2026-09-02

**Authors:** Zhonghui Jin, Pei He, Yangming Guo, Xiaodong Wang, Aiqing Fang

**Affiliations:** 1 School of Software Engineering, Northwestern Polytechnical University, Xi’an 710072, China; jinzhonghui@mail.nwpu.edu.cn; 2 School of Computer Science, Northwestern Polytechnical University, Xi’an 710072, China; hepei002@mail.nwpu.edu.cn; 3 Ningbo Institute of Northwestern Polytechnical University, Ningbo 315103, China; 4 National Center for Applied Mathematics in Chongqing, Chongqing Normal University, Chongqing 401331, China; 5 Chongqing Municipal University Key Laboratory of Optimization Techniques and Intelligent Vehicle, Chongqing 401331, China

**Keywords:** depression recognition, structured prototype learning, temporal sparsity, cross-modal asynchrony

## Abstract

Audio–visual depression recognition in real-world scenarios is often challenged by temporal sparsity and cross-modal asynchrony, where depression-related cues may appear only in short segments and may not align precisely across modalities. Under such conditions, global pooling or dense attention tends to dilute sparse discriminative evidence with redundant context, leading to unstable utterance-level representations. To address this issue, we propose an audio–visual depression recognition framework that integrates modality feature adaptation, bidirectional cross-modal interaction, and graph-based prototype abstraction. Specifically, heterogeneous audio and visual streams are first transformed into compatible representations, after which bidirectional cross-attention models content-dependent dependencies across modalities without requiring index-wise correspondence. The fused tokens are then interpreted as graph nodes and aggregated into a compact set of semantic prototypes through graph convolution and differentiable soft clustering. In addition, audio perturbation is introduced during training as a task-oriented regularisation strategy for partial acoustic evidence loss and temporal misalignment. Experiments on the LMVD dataset demonstrate clear improvements on the primary depression-recognition task, while auxiliary evaluations on MIntRec and CMU-MOSI suggest that the structured prototype representation is beneficial for other temporally sparse audio–visual recognition tasks. These results indicate that structured prototype learning is effective for preserving sparse depression-related cues, while training-time perturbation provides a complementary regularisation effect under asynchronous multimodal conditions.

## 1. Introduction

Depression is a highly prevalent mental disorder that results from the long-term interaction of multiple factors, such as biological genetics, self-awareness, external pressure, and interpersonal conflict, that constitute a major public health burden worldwide [1]. Therefore, identifying depressive states more objectively, automatically, and efficiently in complex, real-world scenes has become a key problem. With the rapid development of machine learning and multimodal affective computing, data-driven depression screening and auxiliary assessment have attracted increasing attention [2]. By using clinical scales, language, video, and other individual multimodal data, an individual’s mental state can be identified, providing a data basis for early screening, risk warning, and personalised intervention for depression. However, building reliable depression recognition models for real-world settings remains difficult, especially when diagnostically relevant cues are sparse, weak, and temporally misaligned across modalities [3].

Researchers have conducted extensive studies on behaviour recognition (e.g., depression recognition), which are primarily categorised into traditional methods [4] and deep learning-based approaches [2]. Traditionally, the diagnosis of depression has relied heavily on clinical interviews and patient self-reports, methods that are inherently subjective and can lead to challenges in accuracy and consistency [5]. These methods often involve standardised psychological scales [6], such as the Centre for Epidemiologic Studies Depression Scale and the Geriatric Depression Scale, to detect symptom severity. The above diagnostic process is effective, but it is labour-intensive, time-consuming, and highly subjective. Therefore, some researchers have attempted to use machine learning to identify patients with depression [7] automatically. These methods typically extract discriminative features through statistical analysis and feature engineering of data from patients with depression and normal individuals. These features are then input into classifiers such as Support Vector Machine (SVM), Random Forest (RF), and Logistic Regression (LR) to automatically determine whether an individual’s mental state is normal [4]. However, traditional methods do not consider individual differences and spatiotemporal characteristics, resulting in weak robustness and applicability of the extracted features, which affects the accuracy of depression recognition. Unlike traditional methods, deep learning-based methods mainly optimise model parameters through data-driven approaches, which offer better robustness and recognition accuracy in complex interactive environments [8]. These methods can be divided into text-based detection methods, audio-based ones, visual-based ones, and physiological signal-based ones [9]. Text modality-based ones mainly extract depression-related vocabulary features and semantic tendencies. Although these methods use convolutional neural networks or Transformers to obtain local and global semantic information, and then emphasise the importance of text segments through bidirectional long short-term memory networks or attention mechanisms, they may lead to potential semantic loss and misreading risks [10]. To this end, further research is being conducted to introduce speech modalities into the field of depression recognition, learning expressions such as emotional dullness, hesitation, and low arousal from prosodic and acoustic features, including fundamental frequency, energy, pace, and pauses [11]. However, speech is highly susceptible to environmental noise, accent, and device differences, resulting in weak model scene generalisation. So, researchers attempted to use visual modalities for multimodal fusion under sparsity and asynchrony, extracting nonverbal behavioural features from individuals’ facial expressions, gaze, head posture, and microexpression dynamics [12]. However, visual cues are easily affected by illumination, occlusion, posture variation, and individual expression control. Physiological signals can provide complementary information related to internal states, but relying on them alone is less suitable for scalable in-the-wild depression recognition because of sensor-dependent acquisition, wearing requirements, and inter-individual baseline variability [13]. These limitations have motivated multimodal approaches that reduce dependence on a single information source by combining complementary evidence across modalities. With deep learning, such complementary information can be learned and integrated directly from heterogeneous representations. For example, previous studies have explored local-to-global multimodal graph modelling [14], multiple cross-attention fusion [15], and dual-stream fusion of EEG and eye-tracking signals [16] to integrate complementary evidence across heterogeneous modalities. Nevertheless, existing multimodal fusion methods still face challenges when informative cues are temporally sparse or asynchronous across modalities.

To address the above issues, this study presents an audio–visual fusion framework based on structured prototype learning. The key idea is that the proposed method constructs a similarity graph over cross-modally enriched fusion tokens and further aggregates them into a compact set of semantic prototypes through differentiable graph clustering. In addition, modality perturbation is introduced during training to simulate common degradation and misalignment conditions, thereby improving the stability of the learned utterance-level representation. In this way, the model can preserve sparse discriminative depressive cues and suppress redundant temporal content, while training-time perturbation regularises representation learning under asynchronous and degraded multimodal conditions. The main contributions of this work are summarised as follows:

- We formulate audio–visual depression recognition under temporal sparsity and cross-modal asynchrony as a multimodal pattern recognition problem, where sparse diagnostic cues should be preserved rather than diluted by global temporal aggregation.
- We propose a structured prototype learning mechanism for sparse audio–visual evidence abstraction. Rather than using graph modelling as a direct cross-modal fusion mechanism, GCFRM operates on cross-modally enriched temporal tokens and organises temporally distributed but semantically related evidence into a compact set of sample-specific latent prototypes before utterance-level aggregation.
- We incorporate audio-focused perturbation during training to expose the model to partial acoustic evidence loss and audio–visual temporal offsets, thereby regularising representation learning under the degraded and asynchronous conditions considered in in-the-wild depression recognition.
- We conduct extensive experiments on LMVD as the primary depression recognition benchmark, and further provide auxiliary transfer evaluations on MIntRec and CMU-MOSI to assess the generality of the learned representation strategy.

The rest of this paper is as follows. Section 2 introduces the related work on multimodal fusion. In Section 3, we introduce the proposed network architecture. In Section 4, we carry out a comparative experiment between our method and the competitors. Then, in Section 5, we conclude the paper and discuss limitations and future work.

## 2. Related Work

Depression detection is a typical application of multimodal behaviour recognition, which can be divided into traditional methods and deep learning-based ones [3].

**Traditional-based Approaches.** Following established clinical criteria, depressive behavioural markers include: (1) reduced vocal energy and prolonged pauses; (2) diminished facial expressivity and eye contact; and (3) psychomotor retardation. Traditional clinical medicine often uses clinical scales to determine whether an individual has depression, e.g., the Hamilton Depression Scale [17], the Zung Self-Rating Depression Scale [18], and the Beck Depression Self-Rating Scale [19]. These methods rely on subjective self-assessment, are time-consuming, and have low accuracy. Therefore, researchers have begun to construct automatic depression recognition models using observable behavioural data such as speech, video, and language. These methods mainly utilise expert experience to design discriminative depression feature operators and feed the extracted features into classifiers, e.g., Support Vector Machine (SVM), k-Nearest Neighbour (KNN), and Decision Trees (DT), to obtain classification results. For example, Wang et al. [20] extracted emotional features from social media texts and proposed a behavioural signal analysis model based on sentiment analysis. Meanwhile, Cohn et al. [21] extracted features such as intensity, duration, and frequency of facial movements from videos and prosodic features from the audio. These manually designed features are used to train classifiers to distinguish between depressed individuals and healthy control groups. Based on facial features, Joshi et al. [22] introduced body posture and movements in the depression classification task to improve the accuracy of depression recognition. However, these methods are sensitive to noise, image quality, and semantic gaps, resulting in poor robustness and generalisation in behavioural signal analysis [23]. Unlike the above methods, some researchers believe that the eyes are the window to the soul and can, to some extent, reflect a person’s mental state. For this purpose, Alghowinem et al. [24] used eye movement features such as fixation, scanning, and blinking for behavioural signal analysis tasks. The recognition of depression based on single-modal data features mentioned above is easily affected by various factors. Therefore, Scherer et al. [25] synthesised features from multiple modalities such as facial action units, head posture, and speech prosody to construct a conditional random field dynamic multimodal fusion model, which significantly improves performance compared with single-modal methods. Although researchers have conducted extensive research on traditional methods for identifying depression, these methods do not consider individual differences and spatiotemporal characteristics, resulting in weak robustness and applicability of the extracted features, which affects the accuracy of depression recognition.

**Deep Learning-based Approaches.** With the development of deep learning technology, new ideas have been proposed to address problems in traditional depression recognition. Many deep learning-based behavioural signal analysis methods have been designed to capture deep semantic features from different modalities through data-driven and learning-based optimisation [2]. These methods can be divided into text-based detection methods, audio-based ones, visual-based ones, physiological signal-based ones, and multimodal data-based ones. For example, Yin et al. [26] proposed a multimodal hierarchical recurrent neural network for depression detection. Daru et al. [27] proposed a transformer-based hybrid architecture to address the dual challenges of modelling long-term temporal dependencies and extracting local acoustic patterns in speech signals. He et al. [28] proposed a resource-adaptive federated domain adaptation method for depression detection in EEG signals. Omar et al. [4] presented a depression classification model based on a large text model, significantly improving depression recognition accuracy. Although these unimodal deep learning models overcome the limitations of traditional methods, they still face challenges in recognition accuracy and robustness. For example, Liu et al. [14] provided a data-driven and reproducible evaluation framework. This method integrates multimodal data such as brain imaging, physiological signals, and behaviour, and evaluates depression severity through graph neural networks. Wang et al. [15] proposed a hybrid model that combines multimodal feature perception and multiple cross-attention fusions. Zhu et al. [16] proposed a dual-stream Transformer architecture that integrates EEG and eye tracking. Related video analysis studies have also explored complementary spatiotemporal feature modelling. Tian et al. [29] exploited illumination-based spatiotemporal inconsistencies for deepfake video detection, while Zhang et al. [30] further combined spatiotemporal illumination features with a convolution-Transformer hybrid architecture. Although these studies address different tasks, they illustrate the value of jointly exploiting complementary local and temporal evidence rather than relying on a single feature representation.

Existing multimodal methods mainly improve heterogeneous feature integration through attention-based fusion, representation disentanglement, or graph-based relational modelling. For example, MISA [31] learns modality-invariant and modality-specific representations, while cross-attention and graph-based approaches exploit interactions or feature relations to enhance multimodal representation learning. Our CFIM builds on the general principle of cross-modal attention rather than treating attention itself as a new mechanism; specifically, it performs bidirectional content-dependent interaction without requiring index-wise correspondence between audio and visual tokens. However, these interaction-oriented approaches primarily focus on modality-level representation learning or cross-modal information exchange, while sparse depression-related evidence may remain distributed across temporally separated segments and be diluted during subsequent global aggregation. Motivated by this limitation, our framework performs cross-modal interaction first and then applies graph-guided prototype abstraction to organise distributed temporal evidence before utterance-level aggregation. Accordingly, the distinction of GCFRM does not lie in graph pooling or prototype learning individually, but in using relation-guided soft grouping after cross-modal interaction to transform distributed temporal evidence into sample-specific latent evidence representations before global aggregation.

## 3. Methodology

Despite recent research progress, many audio–visual fusion solutions are still not well-suited for real-world depression recognition scenarios due to three limitations: (1) temporal sparsity, where discriminative cues are embedded in long non-informative sequences; (2) cross-modal asynchrony, where informative audio and visual cues may occur at different positions and therefore should not be fused under a strict index-wise correspondence assumption; and (3) representation fragility under modality degradation, where unstable acoustic artifacts or temporally misaligned segments may interfere with global representation learning. To address these issues, we propose a structured prototype learning framework that models fused tokens as nodes on a latent similarity graph and abstracts them into compact semantic prototypes through differentiable graph clustering. In addition, we introduce audio perturbation during training to regularise representation learning under simulated degradation and misalignment conditions.

### 3.1. Problem Formulation

Let the training set be denoted by $D={\left\{\left(X_{i}^{\left(v\right)},X_{i}^{\left(a\right)},y_{i}\right)\right\}}_{i=1}^{M},$ where *M* is the number of training samples, $X_{i}^{\left(v\right)}\in R^{T_{v}\times d_{v}}$ is the visual feature sequence, $X_{i}^{\left(a\right)}\in R^{T_{a}\times d_{a}}$ is the audio feature sequence, and $y_{i}\in \left\{0,1\right\}$ denotes the depression label, with 0 and 1 corresponding to normal and depression, respectively. The goal of this work is to learn an utterance-level representation $g_{i}$ that preserves sparse depressive cues under temporal asynchrony and modality degradation. Formally, we define a representation extractor $\Phi_{\Theta }$ and a binary prediction head $h_{\psi }$ as:

$$g_{i}=\Phi_{\Theta }\;\left(X_{i}^{\left(v\right)},X_{i}^{\left(a\right)}\right),\;{\hat{p}}_{i}=h_{\psi }\;\left(g_{i}\right),$$

where Θ and ψ denote the trainable parameters of the representation extractor and prediction head, respectively. During training, the model is optimised by minimising the standard two-class cross-entropy loss:

(1) $$\min_{\Theta ,\psi }-\frac{1}{M}\sum_{i=1}^{M}\sum_{c=0}^{1}I\left(y_{i}=c\right)\log{\hat{p}}_{i,c}.$$

**Figure 1:**
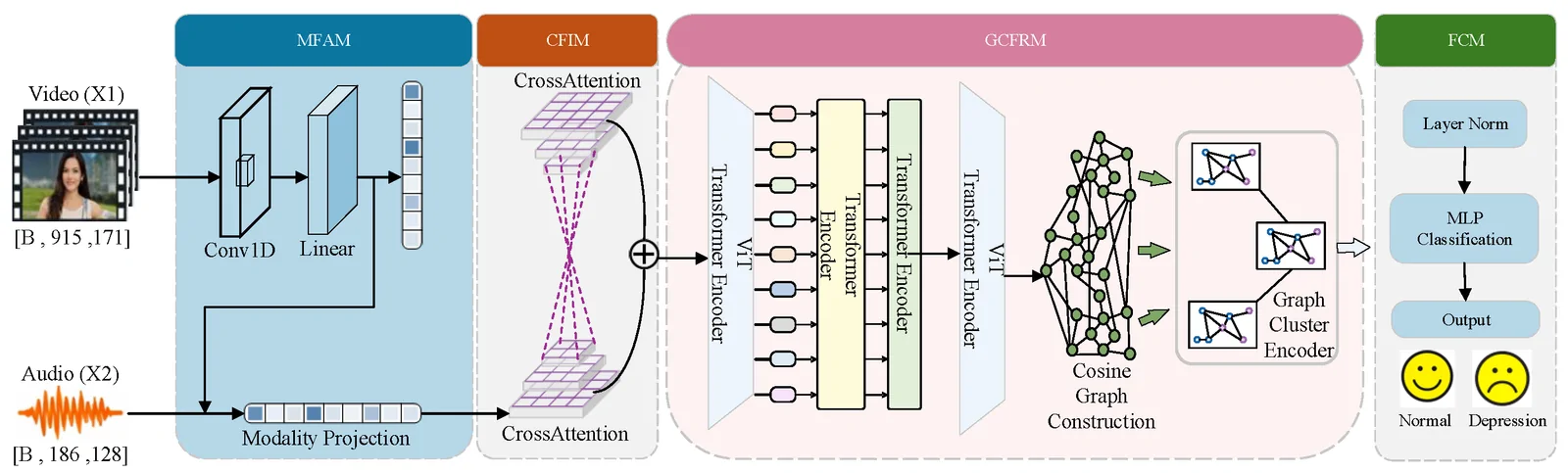
Schematic diagram of structured prototype learning for sparse and asynchronous multimodal fusion.

As shown in Figure 1, the proposed framework consists of three main stages: the Modality Feature Adaptation Module (MFAM), the Cross-Modal Feature Interaction Module (CFIM), and the Graph Clustering Representation Module (GCFRM).

$$\left(X^{\left(v\right)},X^{\left(a\right)}\right)\overset{F_{1}}{\to }\left(T^{\left(v\right)},T^{\left(a\right)}\right)\overset{F_{2}}{\to }H\overset{F_{3}}{\to }g\overset{\mathrm{Head}}{\to }\hat{p},$$

where $F_{1}$, $F_{2}$, and $F_{3}$ denote MFAM, CFIM, and GCFRM, respectively. Head is a classification head that aims to map features to different categories.

### 3.2. Training-Time Audio Perturbation

Audio-based depression recognition relies on behavioural cues such as prosodic variation, speech energy, and pauses, which may be sparsely distributed and are susceptible to recording degradation or local signal loss. In addition, audio and visual behavioural cues may not be temporally synchronised. Accordingly, we perturb only the audio stream during training while keeping the visual stream unchanged: random audio dropout simulates incomplete acoustic evidence, while temporal shifting simulates audio–visual latency. These operations are used as a task-oriented regularisation strategy rather than as new augmentation mechanisms. Let T(·) denote a stochastic perturbation operator applied to the audio stream. The perturbed audio input is written as:

$${\tilde{X}}_{i}^{\left(a\right)}=T\;\left(X_{i}^{\left(a\right)};p,\Delta \right),$$

where *p* denotes the probability of random audio dropout and Δ denotes the temporal shift magnitude. The training objective becomes:

$$\min_{\Theta ,\psi }\frac{1}{M}\sum_{i=1}^{M}E_{{\tilde{X}}_{i}^{\left(a\right)}∼T\left(X_{i}^{\left(a\right)}\right)}\left[L_{\mathrm{ce}}\left(h_{\psi }\;\left(\Phi_{\Theta }\;\left(X_{i}^{\left(v\right)},{\tilde{X}}_{i}^{\left(a\right)}\right)\right),y_{i}\right)\right].$$

Notably, training with these perturbations encourages the model to rely on complementary audio–visual evidence rather than uninterrupted and perfectly synchronised audio.

### 3.3. Modality Feature Adaptation Module

Audio and visual features extracted from different preprocessing pipelines have substantially different sequence sizes and feature dimensions. MFAM is therefore introduced as a representation-adaptation stage rather than an explicit temporal-alignment mechanism. Its purpose is to transform the heterogeneous modality representations into compatible sizes before cross-modal interaction, without assuming that tokens at the same index are temporally or semantically synchronised. For LMVD, the visual input $X^{\left(v\right)}\in R^{915\times 171}$ and the audio input $X^{\left(a\right)}\in R^{186\times 128}$ have different representation sizes. The visual representation is first transformed by a 1D convolution from 915×171 to 186×171, followed by a linear projection from 171 to 128 dimensions:

(2) $${\tilde{X}}^{\left(v\right)}=\mathrm{Linear}\;\left(Conv1D\;\left(X^{\left(v\right)}\right)\right)\in R^{186\times 128}.$$

Since the audio representation already has a size of 186×128, no additional size reduction is applied to the audio stream. Because the visual representation is compressed from 915 input channels to 186 learned channels, some information compression is unavoidable. Since the convolution uses a kernel size of 1 and a stride of 1, this operation should not be interpreted as stride-based temporal subsampling or as a lossless temporal-alignment procedure. Both modality representations are subsequently mapped into the shared latent dimension *d*:

(3) $$T^{\left(v\right)}=\mathrm{Proj}\;\left({\tilde{X}}^{\left(v\right)}\right),\;T^{\left(a\right)}=\mathrm{Proj}\;\left(X^{\left(a\right)}\right),\;T^{\left(v\right)},T^{\left(a\right)}\in R^{186\times d}.$$

The resulting sequences are therefore matched in representation size and latent dimensionality, but this operation should not be interpreted as explicit temporal or semantic synchronisation. Potential correspondence mismatches between the two modalities are subsequently addressed through content-based cross-modal interaction in CFIM.

### 3.4. Cross-Modal Feature Interaction Module

Given the size-matched modality representations $T^{\left(v\right)}$ and $T^{\left(a\right)}$, CFIM performs bidirectional cross-modal interaction to model content-dependent dependencies between audio and visual evidence. Unlike index-wise fusion, cross-attention does not require the *i*-th visual token to correspond directly to the *i*-th audio token, because each query token can attend to all tokens in the complementary modality. Let $\bar{m}$ denote the complementary modality of *m*, i.e., $\bar{v}=a$ and $\bar{a}=v$. The cross-modally enriched representation of modality *m* is defined as:

(4) $${\bar{T}}^{\left(m\right)}=T^{\left(m\right)}+\mathrm{CrossAttn}\;\left(T^{\left(m\right)},T^{\left(\bar{m}\right)},T^{\left(\bar{m}\right)}\right),\;m\in \left\{v,a\right\}.$$

After bidirectional cross-modal interaction, the two modality-specific representations are concatenated and further encoded with positional information:

(5) $$H=\mathrm{Transformer}\;\left(\left[{\bar{T}}^{\left(v\right)};{\bar{T}}^{\left(a\right)}\right]+P\right),$$

where $P\in R^{2N\times d}$ denotes the learnable positional embedding, and $H\in R^{2N\times d}$ is the resulting cross-modally enriched token representation that serves as the input to GCFRM.

### 3.5. Graph Clustering Representation Module

To preserve sparse depression-related cues while suppressing redundant temporal content, GCFRM operates on the cross-modally enriched representation $H\in R^{2N\times d}$ and organises distributed token-level evidence through graph-guided relational modelling and prototype abstraction. Let $h_{1},\ldots ,h_{2N}$ denote the rows of H. Since depression-related behavioural cues may occur at temporally distant positions, we evaluate pairwise affinities among all tokens rather than restricting graph connections to local temporal neighbours. The affinity matrix is defined using rectified cosine similarity:

(6) $${\tilde{A}}_{ij}=\max\left(0,\frac{h_{i}^{⊤}h_{j}}{{∥h_{i}∥}_{2}{∥h_{j}∥}_{2}+\varepsilon }\right)+\delta_{ij},\;1\leq i,j\leq 2N,$$

where $\delta_{ij}$ introduces a self-connection and ε is a small constant for numerical stability. Cosine similarity characterises representation-level affinity while reducing sensitivity to feature magnitude. Because the current GCFRM adopts a nonnegative affinity graph, negative cosine similarities are treated as the absence of positive relations rather than explicitly modelled negative interactions.

The affinity matrix is degree-normalised as:

(7) $${\tilde{D}}_{ii}=\sum_{j=1}^{2N}{\tilde{A}}_{ij},\;\hat{A}={\tilde{D}}^{-\frac{1}{2}}\tilde{A}{\tilde{D}}^{-\frac{1}{2}}.$$

Starting from $Z^{\left(0\right)}=H$, relational information is propagated through residual graph convolution:

(8) $$Z^{\left(\ell +1\right)}=Z^{\left(\ell \right)}+\mathrm{ReLU}\left(\hat{A}Z^{\left(\ell \right)}W_{\mathrm{gcn}}^{\left(\ell \right)}\right),\;\ell =0,\ldots ,L_{g}-1.$$

The graph-enhanced tokens $Z={\left[z_{1},\ldots ,z_{2N}\right]}^{⊤}$ are then organised into *K* sample-specific latent prototypes. The token weights for each prototype are obtained by

(9) $$S={\mathrm{softmax}}_{\mathrm{token}}\left(ZW_{\mathrm{pool}}+b_{\mathrm{pool}}\right),\;S\in R^{2N\times K},$$

where the softmax is applied across the token dimension for each prototype, such that $\sum_{i=1}^{2N}S_{ik}=1$. The *k*-th prototype is therefore

(10) $$c_{k}=\sum_{i=1}^{2N}S_{ik}z_{i},\;k=1,\ldots ,K.$$

Each $c_{k}$ represents a sample-specific latent evidence summary rather than a predefined clinical category or a global class prototype.

Since prototype indices have no predefined ordering, permutation-invariant mean aggregation is used to obtain the utterance-level representation:

(11) $$g=\frac{1}{K}\sum_{k=1}^{K}c_{k}.$$

Thus, GCFRM first models relational dependencies among cross-modally enriched tokens and then summarises distributed evidence into a compact set of latent prototypes. The current implementation uses a dense positive-affinity graph; systematic comparisons with sparsified, thresholded, or signed graph constructions remain for future investigation.

### 3.6. Feature Classification Module (FCM)

After graph prototype aggregation, the utterance-level representation g is fed into a two-layer MLP classifier for final binary depression prediction:

$$u=\mathrm{Dropout}\;\left(\mathrm{GELU}\;\left(W_{1}g+b_{1}\right)\right),$$

$$\hat{p}=\mathrm{softmax}\;\left(W_{2}u+b_{2}\right),$$

where $W_{1}$, $b_{1}$, $W_{2}$, and $b_{2}$ are learnable parameters. The output $\hat{p}=\left[{\hat{p}}_{0},{\hat{p}}_{1}\right]$ denotes the posterior probabilities of the normal and depression classes, respectively. For auxiliary transfer evaluations on other datasets, only the output dimension of the final prediction head is adjusted to match the downstream task.

## 4. Experiments

### 4.1. Experiments Setup

**(1) Metrics.** We use floating-point operations (GFLOPs) and parameter counts (Params) to measure the model’s complexity. We evaluate the depression classification performance using Accuracy, Precision, Recall, F1-score, and Matthews correlation coefficient (MCC). In addition to the conventional classification metrics, MCC accounts for all four entries of the confusion matrix and therefore provides a complementary measure of overall binary classification quality. Accuracy measures overall classification correctness, while Precision and Recall characterise the reliability and coverage of positive predictions, respectively. F1-score provides a harmonic balance between Precision and Recall. MCC additionally incorporates all four entries of the confusion matrix and is therefore used as a complementary measure of binary classification quality, particularly under class imbalance. MCC ranges from −1 to 1, with larger values indicating stronger agreement between predictions and ground-truth labels. For multimodal sentiment analysis on CMU-MOSI, we report 7-class accuracy (Acc._7_) and binary accuracy (Acc._2_) to evaluate classification performance under fine-grained and polarity-based settings, respectively. Additionally, mean absolute error (MAE) is used to assess regression quality.

**(2) Methods.** For the primary LMVD depression-recognition task, we compare the proposed method with four traditional machine learning classifiers (KNN, SVM, LR, and RF) and seven representative deep learning baselines (BiLSTM, Xception, ViT, SEResnet, MDDformer, INMS, and DepMamba). These methods cover conventional hand-crafted feature pipelines, temporal sequence models, visual backbones, transformer-based models, and recent multimodal depression recognition approaches. For MIntRec and CMU-MOSI, the reported results are included as auxiliary cross-task references to examine the applicability of the proposed representation mechanism beyond depression recognition. These comparisons should not be interpreted as strictly modality-controlled benchmarks, since the reported baseline configurations may differ in their input modalities and evaluation implementations, with some methods additionally incorporating textual information.

**(3) Dataset.** We primarily evaluate the proposed method on LMVD for depression recognition and use MIntRec and CMU-MOSI as auxiliary datasets to examine the cross-task applicability of the learned representation mechanism. (1) The LMVD [32] is a large-scale multimodal vlog dataset designed for depression detection in the wild. (2) MIntRec [33] is a benchmark dataset for multimodal intent recognition. (3) CMU-MOSI [34] is a benchmark dataset widely used for multimodal sentiment analysis. For LMVD, we follow the sample-level 10-fold cross-validation setting used in the released benchmark implementation. Specifically, each processed video feature file is treated as one sample, and stratified 10-fold cross-validation is performed according to the depression label with shuffling enabled and a fixed random state of 42. In each fold, the held-out samples are used exclusively for testing, while the remaining samples are used for training. Thus, the same video sample never appears in both the training and test sets within a fold. We emphasise that this protocol is performed at the video/sample level rather than at the participant level. Therefore, the reported LMVD results should be interpreted as performance under the sample-level benchmark protocol rather than as a strictly participant-independent evaluation. Since LMVD contains 1823 video samples from 1475 participants, sample-level partitioning does not by itself guarantee that different videos from the same participant are assigned to the same fold. Accordingly, participant-level identity overlap cannot be strictly excluded under the current evaluation protocol.

**(4) Implementation details.** We conduct all experiments on a Windows 10 device equipped with an Intel(R) Xeon(R) w5-3435X 3.10 GHz CPU, 256 GB of RAM, and a single NVIDIA RTX A5000 GPU with 32 GB of memory. The proposed model and all baselines are implemented in Python 3.9 using the PyTorch 1.13.0 deep learning framework with CUDA acceleration. To optimise the model parameters, we adopt the cross-entropy loss and the Adam optimiser. We train the model and evaluate its performance on the LMVD [32], MIntRec [33] and CMU-MOSI [34] datasets for three tasks.

In the experiments, the number of attention heads is set to 2, the network depth is 8, and the number of training epochs is 200. The number of latent prototypes in GCFRM is fixed to K=4 throughout the main experiments. On LMVD, the visual representation is obtained by concatenating four types of behavioural features, including FAUs, facial landmarks, eye gazes, and head pose, resulting in a 171-dimensional feature vector. Audio features are extracted using the pre-trained VGGish model, yielding a 128-dimensional representation. After feature extraction, no additional feature-wise normalisation or value-imputation procedure is applied. Variable-length sequences are handled by truncation when necessary and zero padding for shorter sequences, producing visual and audio inputs of sizes 915×171 and 186×128, respectively. For MFAM, the visual input is mapped from 915×171 to 186×171 using a 1D convolution with 915 input channels, 186 output channels, a kernel size of 1, and a stride of 1, followed by a linear projection from 171 to 128 dimensions. Since the audio input already has size 186×128, no additional temporal compression is applied to the audio stream. Both streams are subsequently projected to the shared latent dimension d=256, resulting in N=186 tokens for each modality.

### 4.2. Comparative Experiment

**(1) Quantitative Comparison of Depression Recognition.** As shown in Table 1, the proposed method achieves the highest reported mean values for Accuracy, Recall, and F1-score among the compared methods on LMVD. Relative to the best traditional classifier, RF, the proposed model improves Accuracy, Precision, Recall, and F1-score by 9.38, 8.70, 10.94, and 9.69 percentage points, respectively. Compared with MDDformer, the proposed method achieves higher reported mean values by 1.73, 1.02, 3.29, and 2.01 percentage points in Accuracy, Precision, Recall, and F1-score, respectively. Since the improvement in mean Accuracy is smaller than the observed cross-fold variability, these differences are interpreted as descriptive performance gains rather than evidence of statistically significant superiority.

**(2) Auxiliary Cross-Task Evaluation on MIntRec.** We use MIntRec as an auxiliary task to examine whether the proposed representation mechanism remains applicable to multimodal intent recognition. As shown in Table 2, the proposed method achieves an accuracy of 73.24% and an F1-score of 70.57%. It obtains the highest accuracy among the reported methods but does not uniformly outperform the compared baselines in Precision, Recall, or the average score. Therefore, the MIntRec results are reported only as auxiliary cross-task evidence that the proposed representation can be applied to a different multimodal recognition task, rather than as evidence of overall superiority or state-of-the-art performance.

**(3) Auxiliary Cross-Task Evaluation on CMU-MOSI.** We further evaluate the proposed representation mechanism on CMU-MOSI as an auxiliary multimodal sentiment-recognition task. As shown in Table 3, the proposed audio–visual framework obtains an Acc.7 of 46.2%, an Acc.2 of 85.4%, an F1-score of 85.3%, and an MAE of 0.7156, indicating that the structured representation mechanism remains applicable beyond the primary depression-recognition task. However, the reported baseline results are not strictly modality-matched to our setting. In particular, some CMU-MOSI baselines incorporate textual information in addition to audio and visual signals. Consequently, Table 3 is intended to provide contextual cross-task evidence rather than a controlled comparison of model superiority.

### 4.3. Analysis of Experimental Results

#### 4.3.1. Effectiveness Analysis of Tested Modules and Training Perturbation

**Figure 2:**
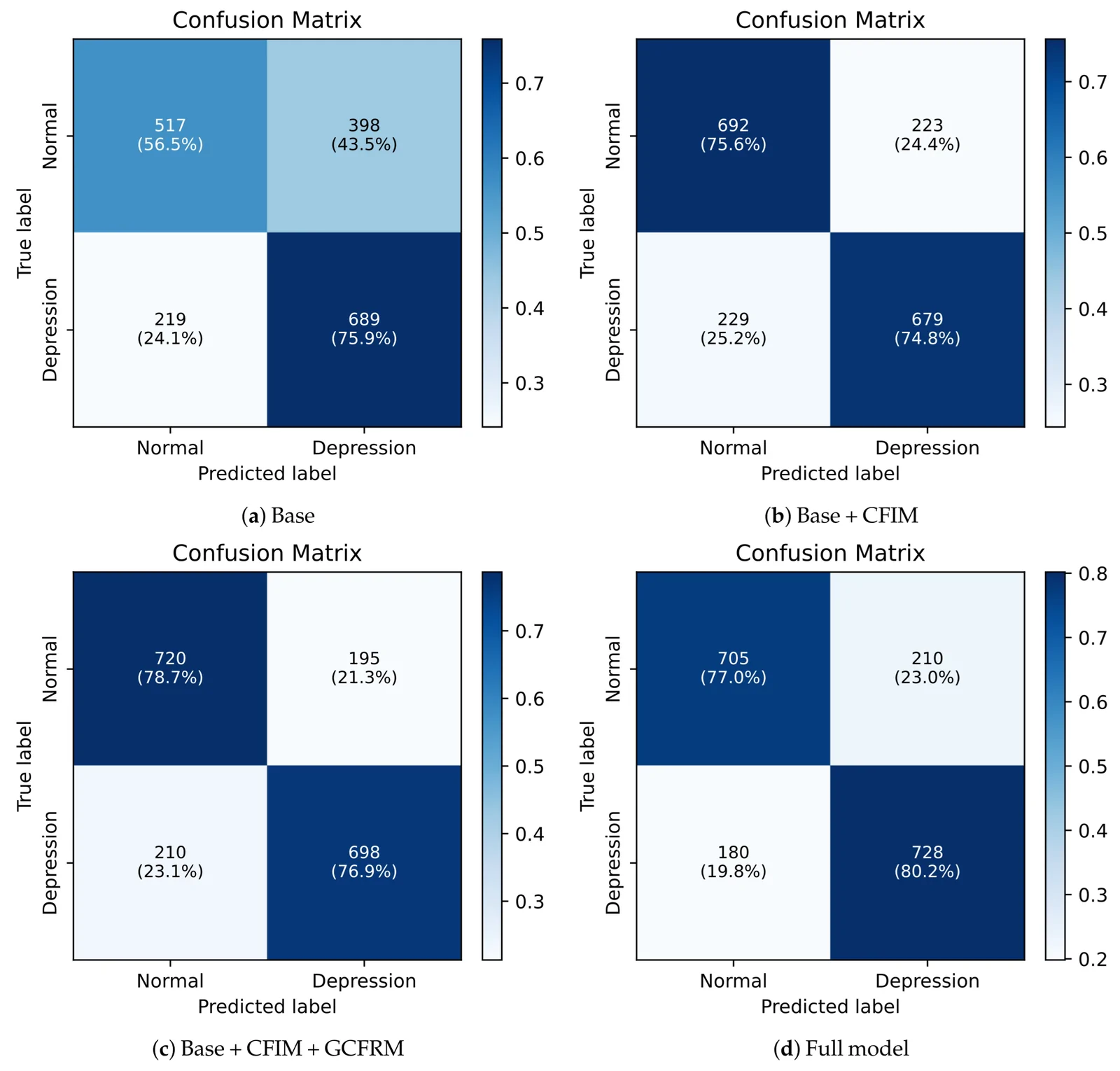
Confusion matrices constructed from the aggregated held-out predictions across the 10 cross-validation folds for the step-wise ablation study on LMVD: (**a**) Base, (**b**) Base + CFIM, (**c**) Base + CFIM + GCFRM, and (**d**) Full model with training-time perturbation.

To evaluate the contribution of the tested components, we conduct a step-wise ablation study, with the results reported in Figure 2 and Table 4. The baseline consists of MFAM and the classification head. We then progressively add the CFIM, GCFRM, and finally the training-time audio perturbation strategy. As shown in Figure 2 and Table 4, adding CFIM markedly improves the representation quality over the baseline, increasing accuracy from 66.16% to 75.21% and indicating that bidirectional cross-modal interaction is critical for modelling complementary audio–visual cues. After further introducing GCFRM, the accuracy rises from 75.21% to 77.78%, and the F1-score improves from 74.85% to 77.44%, showing that graph-guided prototype abstraction helps preserve sparse discriminative evidence before utterance-level aggregation. Compared with Base + CFIM + GCFRM, the full model (Base + CFIM + GCFRM + Perturbation) further improves Accuracy from 77.78% to 78.61% and F1-score from 77.44% to 78.86%, corresponding to gains of 0.83 and 1.42 percentage points, respectively. Because the held-out samples are evaluated without additional corruption, this improvement is interpreted primarily as a regularisation effect of the training-time perturbation rather than as direct evidence of robustness to test-time degradation. By exposing the model to partially missing or temporally displaced acoustic evidence during training, the perturbation discourages excessive reliance on uninterrupted and perfectly synchronised audio. It encourages the model to exploit complementary audio–visual cues, which can improve generalisation to the clean held-out samples. To further assess binary classification quality using all four entries of the confusion matrix, we additionally calculate MCC from the aggregated held-out predictions shown in Figure 2. The MCC increases from 0.3300 for Base to 0.5041 after introducing CFIM, further reaches 0.5557 with GCFRM, and achieves 0.5725 for the full model. This consistent improvement indicates that the gains observed in Accuracy and F1-score are accompanied by a more balanced improvement across the confusion-matrix components.

#### 4.3.2. Analysis of Tested Fusion Strategies

**Figure 3:**
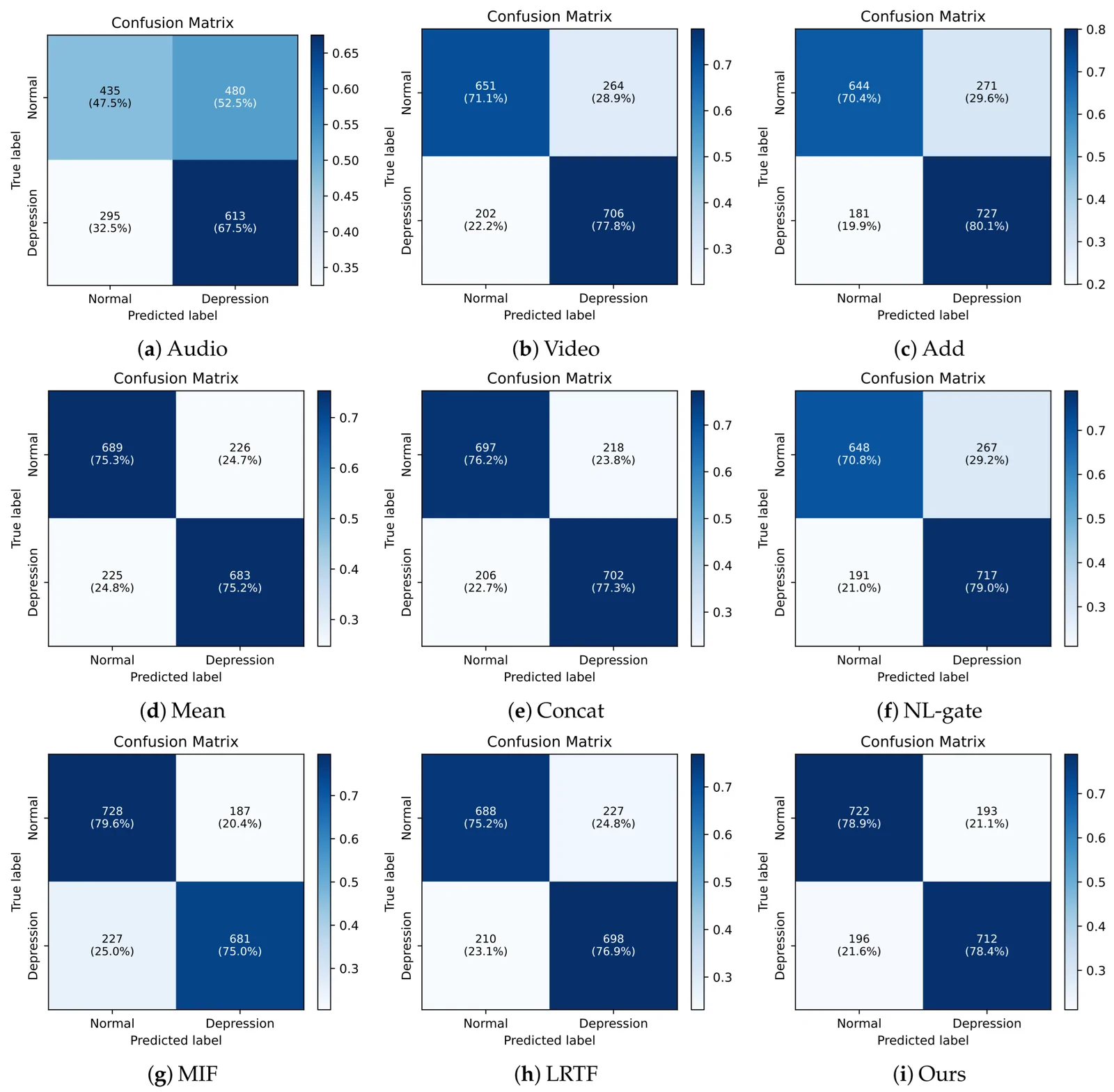
Confusion matrices of different fusion strategies: Audio, Video, Add, Mean, Concat, NL-gate, MIF, LRTF, and Ours.

This subsection analyses the influence of different fusion strategies on depression detection. Specifically, we evaluate nine settings: element-wise addition (Add), element-wise averaging (Mean), channel concatenation (Concat), non-local gating mechanism (NL-gate), Multiplicative Interactions Fusion (MIF), and Low-Rank Tensor Fusion (LRTF), compared against single-modal baselines (audio-only, video-only) and the proposed method (Ours). We report Accuracy (Acc.), Precision (Prec.), Recall, F1-score, and their average (Avg.) as performance metrics, together with inference time (Time, ms), computational complexity (FLOPs, G), and parameter count (Params, M) to assess predictive performance and efficiency. The comparative results are illustrated in Figure 3 and Table 5.

The quantitative results show that the video-only model outperforms the audio-only model under the evaluated setting, indicating that visual behavioural cues provide stronger discriminative evidence in this comparison. Multimodal fusion generally yields higher classification performance than either single-modality baseline. Among the tested fusion operators, Concat achieves higher Accuracy than Add (76.75% versus 75.21%), suggesting that concatenation better preserves modality-specific information before subsequent interaction. In contrast, element-wise addition directly superposes the two modality representations and may partially obscure modality-specific differences.

#### 4.3.3. Stability Analysis via 10-Fold Cross-Validation

This subsection analyses the stability of the proposed method under the sample-level 10-fold cross-validation protocol on LMVD. The 1823 video samples are stratified into ten mutually exclusive folds according to their depression labels, with each fold used once as the held-out test set and the remaining nine folds used for training.

**Figure 4:**
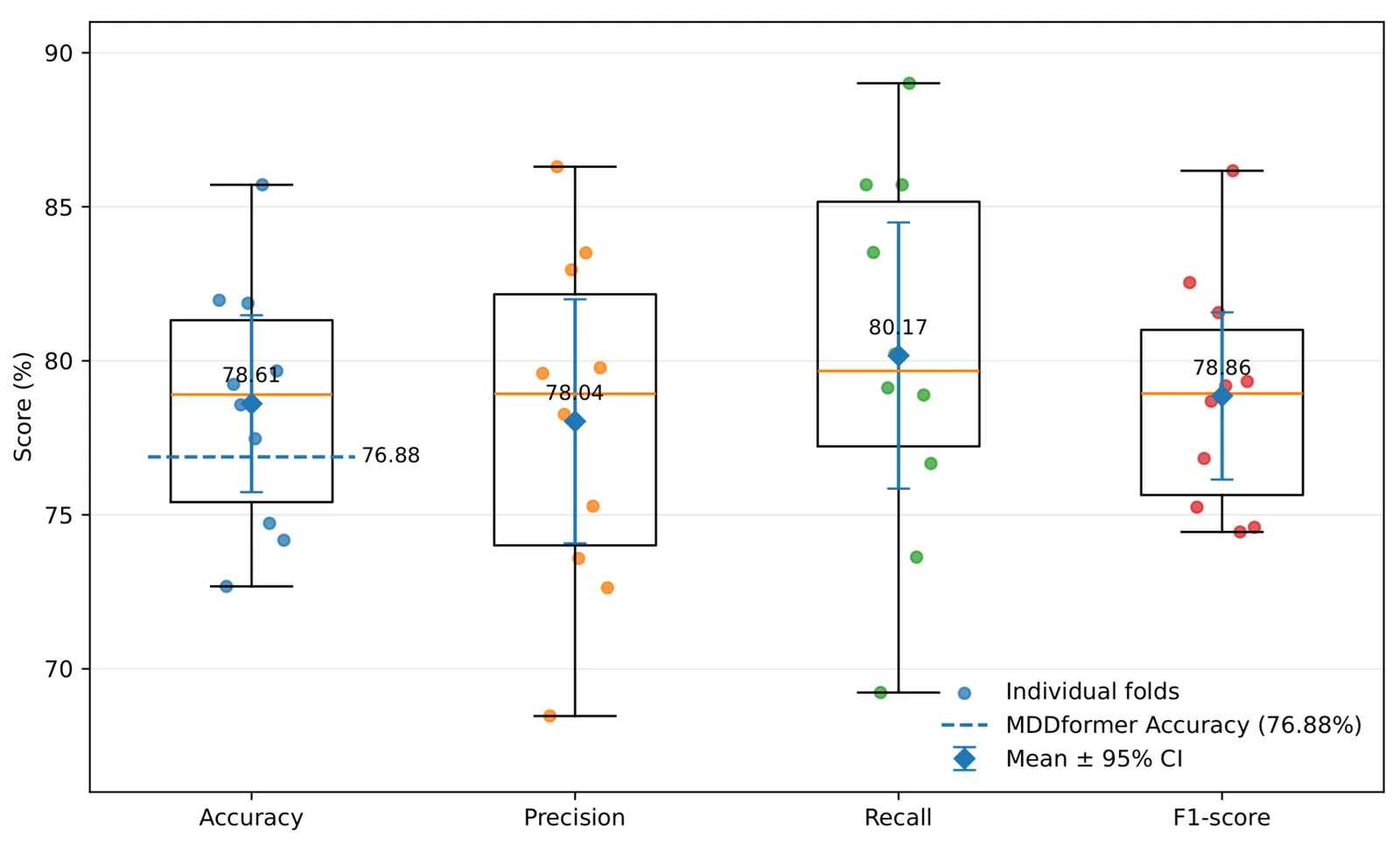
Cross-fold stability of the full model on LMVD. Boxplots summarise the distributions of Accuracy, Precision, Recall, and F1-score across the 10 held-out folds, while individual points denote fold-wise results. The different colors of the individual points are used only for visual distinction, and the orange horizontal lines inside the boxes denote the median values. Diamond markers and error bars indicate the mean values and corresponding 95% confidence intervals. The reported MDDformer Accuracy of 76.88% is shown only as a reference for the Accuracy comparison.

Figure 4 presents the cross-fold performance distribution of the full model under the sample-level 10-fold cross-validation protocol on LMVD. The mean Accuracy, Precision, Recall, and F1-score values are 78.61%, 78.04%, 80.17%, and 78.86%, respectively, with corresponding standard deviations of 4.02%, 5.54%, 6.04%, and 3.79%. The individual fold observations and boxplots indicate moderate cross-fold variability, with F1-score and Accuracy showing relatively smaller dispersion than Precision and Recall. To examine the reported Accuracy difference relative to MDDformer, we additionally performed a two-sided one-sample reference-value test using the reported MDDformer Accuracy of 76.88%. The test yields t(9)=1.36 and p=0.207. This reference-value test is provided only as a supplementary check. It does not replace a paired comparison between the two methods, which would require fold-wise MDDformer results obtained using identical data partitions. Therefore, the observed difference in mean Accuracy is interpreted as a descriptive performance improvement rather than evidence of statistical superiority. This analysis evaluates sensitivity to sample partitioning and should not be interpreted as evidence of participant-independent generalisation.

### 4.4. Training-Time Perturbation Sensitivity Analysis

**Figure 5:**
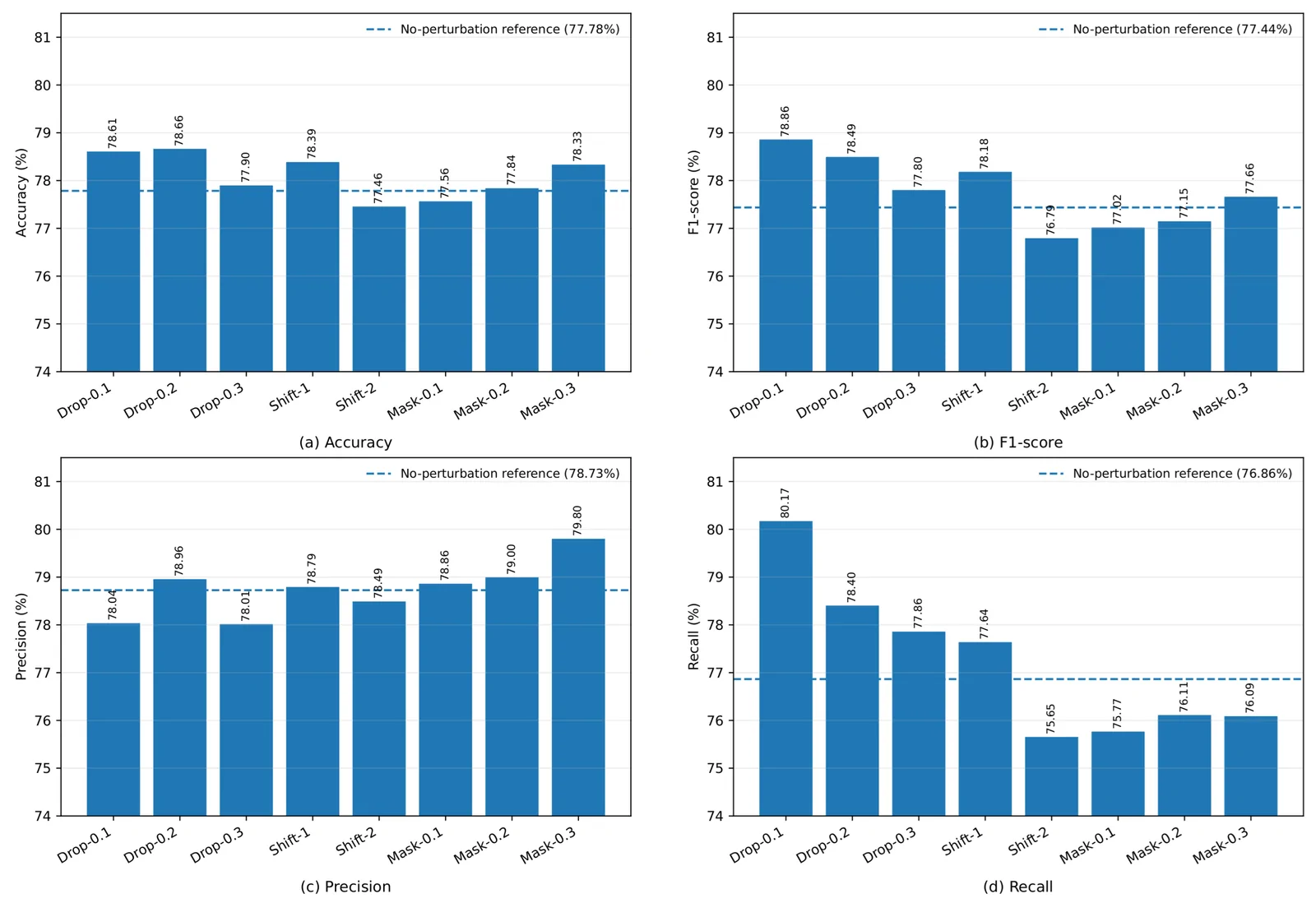
Performance under different training-time perturbation configurations. Bars represent the results obtained under each discrete perturbation setting, while the horizontal dashed line denotes the unperturbed reference using the full architecture without additional training-time perturbation. All results are obtained under the same sample-level 10-fold cross-validation protocol.

**Figure 6:**
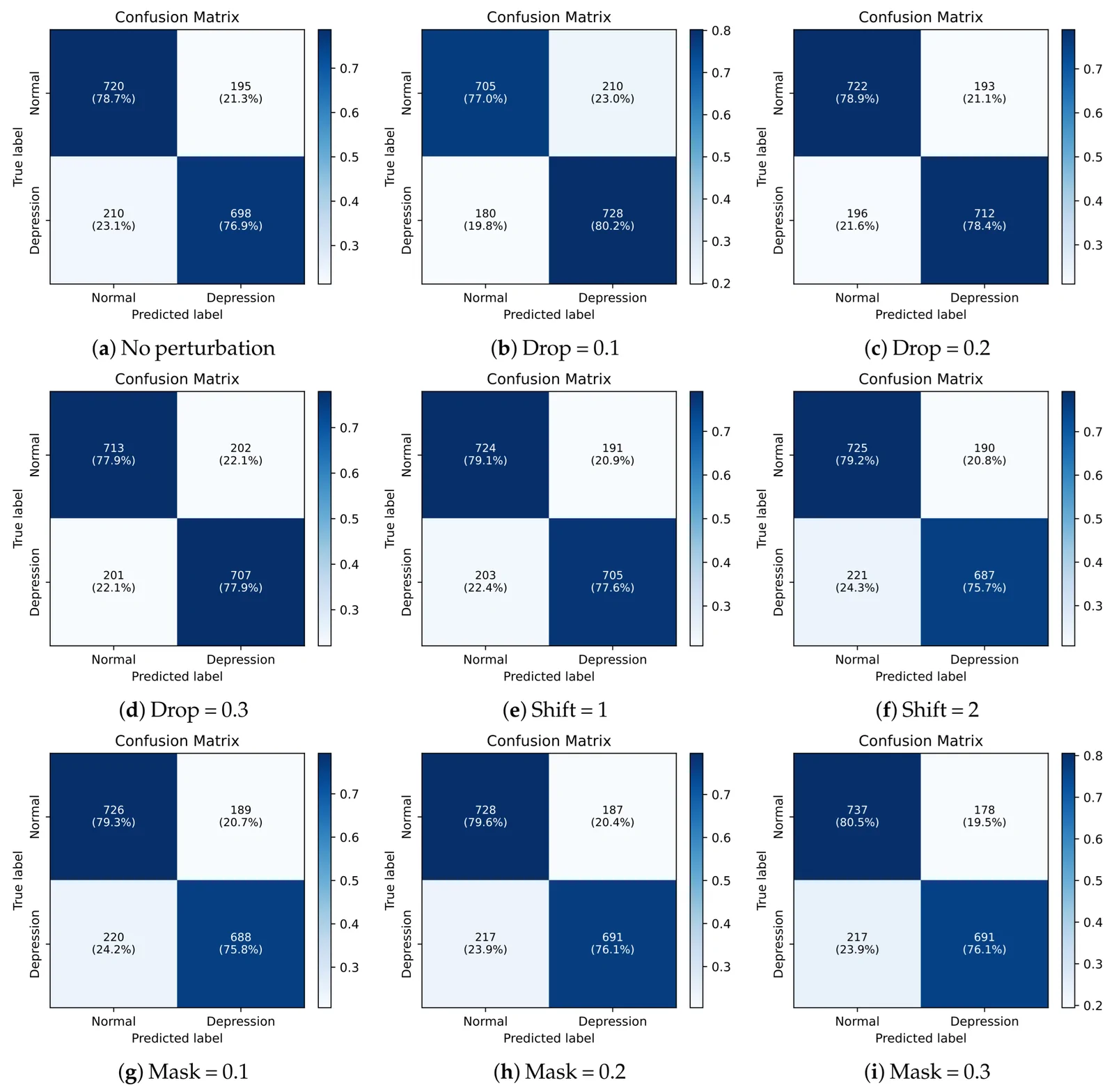
Confusion matrices constructed from the aggregated held-out predictions across the 10 folds under different training-time perturbation configurations.

To examine the sensitivity of the proposed framework to different training-time perturbation configurations, we compare the full architecture under the same 10-fold cross-validation protocol. The full architecture without additional training-time perturbation (i.e., Base + CFIM + GCFRM) is used as the unperturbed reference. For audio dropout, the severity $r_{d}\in \left\{0.1,0.2,0.3\right\}$ denotes the proportion of audio feature segments removed during training. For temporal shifting, Δ∈{1,2} denotes a displacement of the audio sequence by one or two token positions relative to the visual sequence during training. For masked-noise perturbation, $r_{n}\in \left\{0.1,0.2,0.3\right\}$ denotes the proportion of audio segments replaced by zero-mean Gaussian noise during training. Each configuration is evaluated using the same sample-level 10-fold cross-validation protocol with a fixed random-seed setting. Independent repeated evaluations with multiple perturbation seeds are not performed. Therefore, the results are interpreted as sensitivity to different training-time perturbation configurations rather than as a statistical estimate of variability across independent perturbation realisations. Figure 5 compares the different training-time perturbation configurations using bar charts. Because the tested perturbation severities are discrete experimental settings rather than samples from a continuous variable, the bar representation provides a more appropriate comparison than the previous line plots. The performance of the full architecture without training-time perturbation is additionally shown as a horizontal dashed reference line in each panel. Mild audio dropout ($r_{d}=0.1$) improves several metrics relative to the unperturbed reference, suggesting a possible regularisation effect, whereas larger dropout ratios do not provide a consistent additional benefit. Increasing the temporal shift from one to two token positions produces a more evident decrease in Recall, indicating greater sensitivity to stronger audio–visual asynchrony. The masked-noise configurations result in comparatively moderate performance variations. Figure 6 further presents the corresponding confusion matrices aggregated from the held-out predictions across the ten folds. These results characterise sensitivity to different training-time perturbation configurations and should not be interpreted as robustness to test-time corruption.

### 4.5. Performance-Complexity Trade-Off Analysis

**Figure 7:**
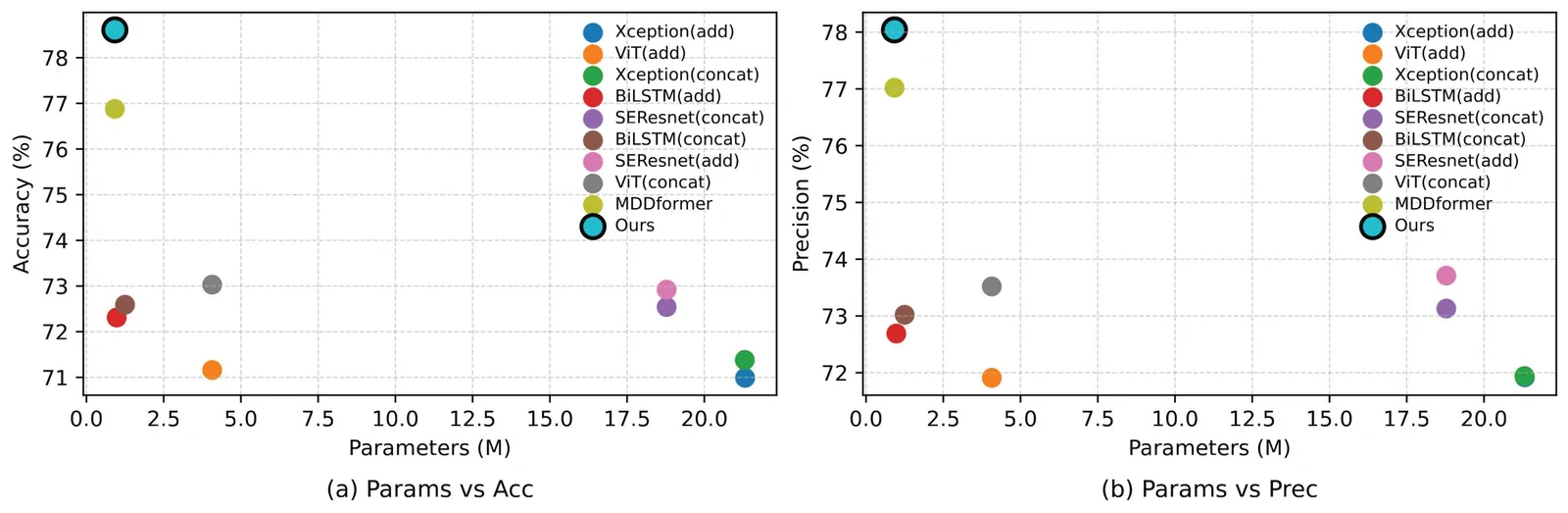

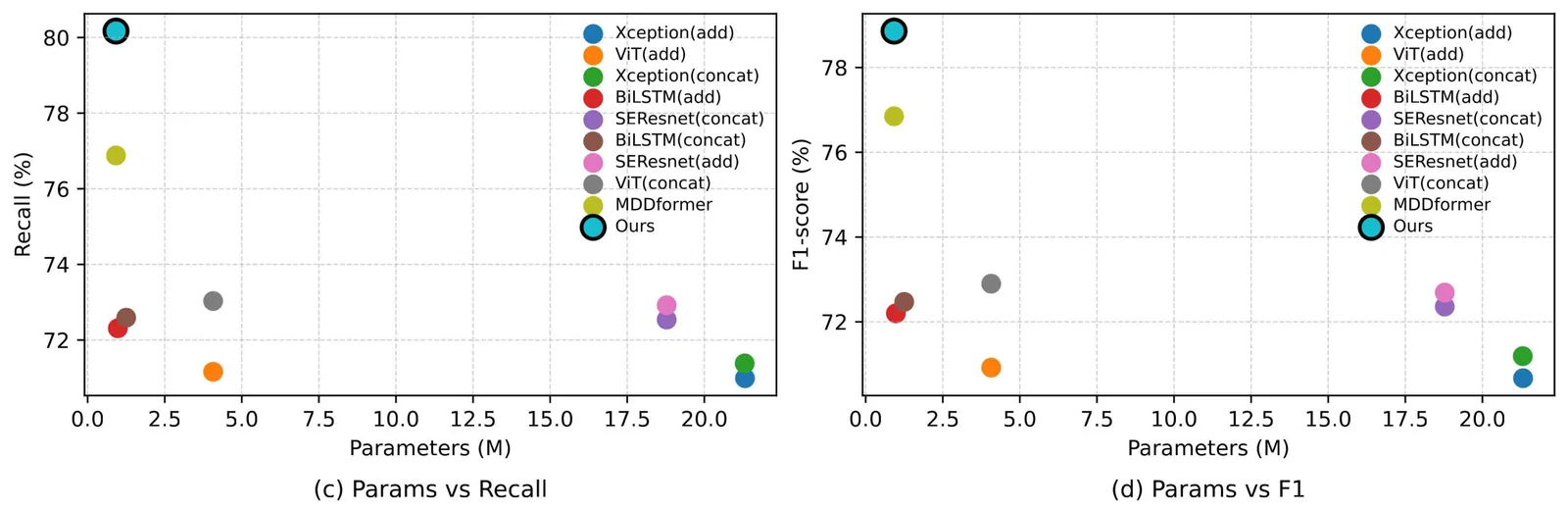
Efficiency analysis. In subfigure (**b**), the marker of Xception(add) overlaps with that of Ours and is therefore not separately visible. Subfigures (**a**) and (**d**) present Accuracy and F1-score, respectively; although their distributions appear similar, they represent different evaluation metrics and are therefore both retained.

We further analyse the trade-off between predictive performance and computational cost on LMVD. As shown in Table 5 and Figure 7, the proposed method achieves the best accuracy (78.61%) among the compared fusion strategies, while incurring higher inference time (5.06 ms) and FLOPs (0.89 G) than several simpler fusion operators such as Add, Concat, and NL-gate. Therefore, the advantage of our method is not minimum latency, but the best predictive performance under a still lightweight parameter budget. These results suggest that the proposed model offers a favourable performance-complexity trade-off. In other words, it improves recognition quality substantially without introducing prohibitive computational overhead, which makes it suitable for deployment scenarios where accuracy is prioritised but computational resources remain limited.

## 5. Conclusions

This paper investigated audio–visual depression recognition from the perspective of sparse and asynchronous multimodal pattern representation. By treating multimodal temporal tokens as graph-structured observations rather than independent sequence elements, the model can capture relational dependencies among sparse discriminative cues. In addition, the training-time perturbation strategy discourages excessive reliance on uninterrupted, perfectly synchronised audio and acts as a task-oriented regulariser during training by exposing the model to simulated degradation and temporal misalignment. Experiments on MIntRec and CMU-MOSI provide preliminary evidence that the proposed representation mechanism remains applicable to other multimodal recognition tasks. These results are intended as cross-task evidence rather than direct generalisation from depression recognition or strictly modality-controlled superiority over the reported baselines. Despite these advantages, this work still has several limitations. First, the current LMVD evaluation employs a sample-level rather than participant-level 10-fold cross-validation protocol, and participant-level identity overlap across folds cannot be strictly ruled out. Consequently, the reported LMVD results should not be interpreted as evidence of participant-independent generalisation. Larger multi-centre clinical datasets and participant-level evaluation are required to further validate generalisation across populations, recording environments, and diagnostic protocols. Second, the current framework focuses exclusively on audio–visual signals and does not explicitly incorporate text, physiological signals, or clinical-scale information, which may provide complementary evidence for depression assessment. Third, although the learned prototypes enhance recognition performance, their clinical interpretability requires further investigation. Future work will extend the proposed method to missing-modality and multi-source settings by incorporating text and physiological signals to improve the reliability of audio–visual depression recognition in practical screening and decision-support applications. In addition, the current study uses a fixed number of prototypes (K=4), and a systematic analysis of prototype granularity across different datasets remains an important direction for future work.

## Figures and Tables

**Table 1:** Comparison with Baseline Methods on the LMVD Dataset (%). Bold indicates the best results.

Model	Acc.	Prec.	Recall	F1	Params	FLOPs
KNN	58.34	59.75	58.34	56.87	–	–
SVM	64.66	65.77	64.66	64.06	–	–
LR	64.88	65.19	64.88	64.73	–	–
RF	69.23	69.34	69.23	69.17	–	–
Xception(add)	70.99	71.92	70.99	70.67	21.32 M	0.46 G
ViT(add)	71.16	71.91	71.16	70.92	4.07 M	0.26 G
Xception(concat)	71.38	71.94	71.38	71.19	21.31 M	0.78 G
BiLSTM(add)	72.31	72.69	72.31	72.20	0.98 M	0.72 G
SEResnet(concat)	72.54	73.13	72.54	72.36	18.78 M	0.96 G
BiLSTM(concat)	72.59	73.02	72.59	72.47	1.25 M	0.92 G
SEResnet(add)	72.92	73.71	72.92	72.69	18.78 M	0.55 G
ViT(concat)	73.03	73.52	73.03	72.90	4.07 M	0.39 G
MDDformer [32]	76.88	77.02	76.88	76.85	0.92 M	0.62 G
INMS [35]	74.34	78.80	75.29	77.06	38.31 M	83.16 G
DepMamba [36]	72.13	70.18	76.56	73.20	6.78 M	13.31 G
**Ours**	**78.61**	**78.04**	**80.17**	**78.86**	2.64 M	0.89 G

**Table 2:** Auxiliary cross-task results on the MIntRec dataset (%). Reported baseline results are provided as contextual references rather than as a strictly modality-controlled comparison. Bold indicates the best results.

Type	Acc.	Prec.	Recall	F1	Avg.
MAG-BERT [37]	70.34	68.31	69.36	68.19	69.05
MulT [38]	72.58	70.73	69.47	69.36	70.54
MISA [31]	72.36	71.24	70.41	70.57	71.15
EMOE [39]	72.58	72.08	70.86	70.73	71.56
**Ours**	**73.24**	**69.58**	**69.64**	**70.57**	**70.76**

**Table 3:** Auxiliary cross-task results on the CMU-MOSI dataset. Reported baseline results are provided for contextual reference; modality configurations are not identical across all methods, and some baselines additionally use textual information. Bold indicates the best results.

Methods	Acc._7_	Acc._2_	F1	MAE
EF-LSTM [40]	33.7	75.3	75.2	1.386
LMF [41]	36.9	78.7	78.7	0.931
MFN [42]	35.6	78.4	78.4	0.964
MulT [38]	35.1	80.0	80.1	0.936
MISA [31]	41.8	84.2	84.2	0.754
DMD [43]	46.2	83.2	83.2	0.721
**Ours**	**46.2**	**85.4**	**85.3**	**0.7156**

**Table 4:** Performance comparison of different modules and training perturbation (%). Bold indicates the best results.

Modules	Acc.	Prec.	Recall	F1	Avg.
Base	66.16	63.83	75.89	68.97	68.71
Base + CFIM	75.21	75.66	74.77	74.85	75.12
Base + CFIM + GCFRM	77.78	78.73	76.86	77.44	77.70
Base + CFIM + GCFRM + Per.	**78.61**	78.04	**80.17**	**78.86**	**78.92**

**Table 5:** Classification performance of different fusion strategies (drop_audio = 0), i.e., Add, Mean, Concat, NL-gate, Multiplicative Interactions Fusion (MIF), Low-Rank Tensor Fusion (LRTF), and Ours (%).

Methods	Acc.	Prec.	Recall	F1	Avg.	Time	FLOPs	Params
audio	57.49	56.57	67.55	60.35	62.29	4.97	0.47	2.60
video	74.44	73.05	77.75	75.00	75.06	4.27	0.47	2.60
Add	75.21	75.21	80.07	76.25	76.68	3.69	0.47	2.60
Mean	75.26	75.36	75.20	75.06	75.22	4.98	0.47	2.60
Concat	76.75	76.52	77.32	76.58	76.79	3.73	0.49	2.73
NL-gate	74.88	73.75	78.97	75.61	75.80	2.73	0.49	2.73
MIF	77.29	78.44	75.00	77.44	76.59	3.69	0.47	2.60
LRTF	76.03	75.83	76.88	76.06	76.20	4.75	0.47	2.62
Ours	78.61	78.04	80.17	78.86	78.92	5.06	0.89	2.64

## Data Availability

The code and processed data for this study are available at: https://github.com/SalvaFang (will be made publicly available upon acceptance), (accessed on 23 August 2026).
